#  Reversible Pisa syndrome caused by chronic subdural hematoma in a patient with Parkinson’s disease: a case report

**DOI:** 10.1186/s12883-022-02972-z

**Published:** 2022-11-15

**Authors:** Eriko Igami, Motoki Fujimaki, Mai Shimizu, Yuta Ishiguro, Takuma Kodama, Yasuyuki Okuma, Nobutaka Hattori, Kazuyuki Noda

**Affiliations:** 1 Department of Neurology, Juntendo University Shizuoka Hospital, 1129, 410-2295 Nagaoka, Izunokuni, Shizuoka Japan; 2 Department of Neurology, Juntendo University School of Medicine, 2-1-1 Hongo, Bunkyo, 113-8421 Tokyo, Japan; 3 Department of Neurosurgery, Juntendo University Shizuoka Hospital, 1129, 410-2295, Nagaoka, Izunokuni, Shizuoka Japan

**Keywords:** Parkinson’s disease, Pisa syndrome, Chronic subdural hematoma, Body schema, Frontal lobe

## Abstract

**Background:**

Pisa syndrome (PS), characterized by lateral trunk flexion, is quite common in patients with Parkinson’s disease (PD). Patients with PS are older and have a significantly longer disease duration, more severe motor phenotype, ongoing combined treatment with levodopa and dopamine agonists, and higher levodopa equivalent daily dose. We describe here, to the best of our knowledge, the first case of a woman with PD who developed acute-onset PS caused by chronic subdural hematoma (CSDH).

**Case presentation:**

A 70-year-old woman developed acute-onset lateral flexion of her trunk to the left side while standing, and she was admitted to our hospital. One month before, she had a mild head trauma with loss of consciousness. At 65 years of age, she noticed difficulty with walking and clumsiness with her hands. She was diagnosed as having PD (Hoehn and Yahr stage 2) and levodopa was initiated. Her symptoms were markedly improved. At 67 years of age, she developed orthostatic hypotension and was treated sequentially with fluids, compression stockings, and midodrine. Urgently performed brain computed tomography (CT) showed a CSDH in the right hemisphere resulting in a marked compression of the hemisphere. After surgical evacuation, her PS disappeared. She has fully recovered to her preoperative level of function.

**Conclusion:**

The present case provides a valuable insight, that is, the mesial frontal lobe and its connections from the posterior parietal cortex play crucial roles in maintaining the body schema and in the pathophysiology of PS. This case suggests that CSDH should be considered when clinicians examine acute-onset PS, even in patients with neurodegenerative disorders such as PD. Appropriate patient triage and timely neurosurgical intervention should be considered.

## Background

Pisa syndrome (PS) is characterized by a marked lateral deviation of the trunk, which resolves or substantially improves in the supine position. PS is usually observed in patients with Alzheimer’s disease treated with a cholinesterase inhibitor, dementia with Lewy bodies, Parkinson’s disease (PD), or atypical parkinsonism such as multiple system atrophy [1, 2]. Interestingly, PS has also been recognized in neurological diseases including normal-pressure hydrocephalus, cerebral infarction, and subdural hematoma [2–4]. Here, we report the case of a PD patient who developed acute-onset lateral flexion of her trunk to the left side, i.e., PS caused by chronic subdural hematoma (CSDH). To the best of our knowledge, cases of PD patients with PS caused by CSDH have not been reported.

## Case presentation

**Fig. 1:**
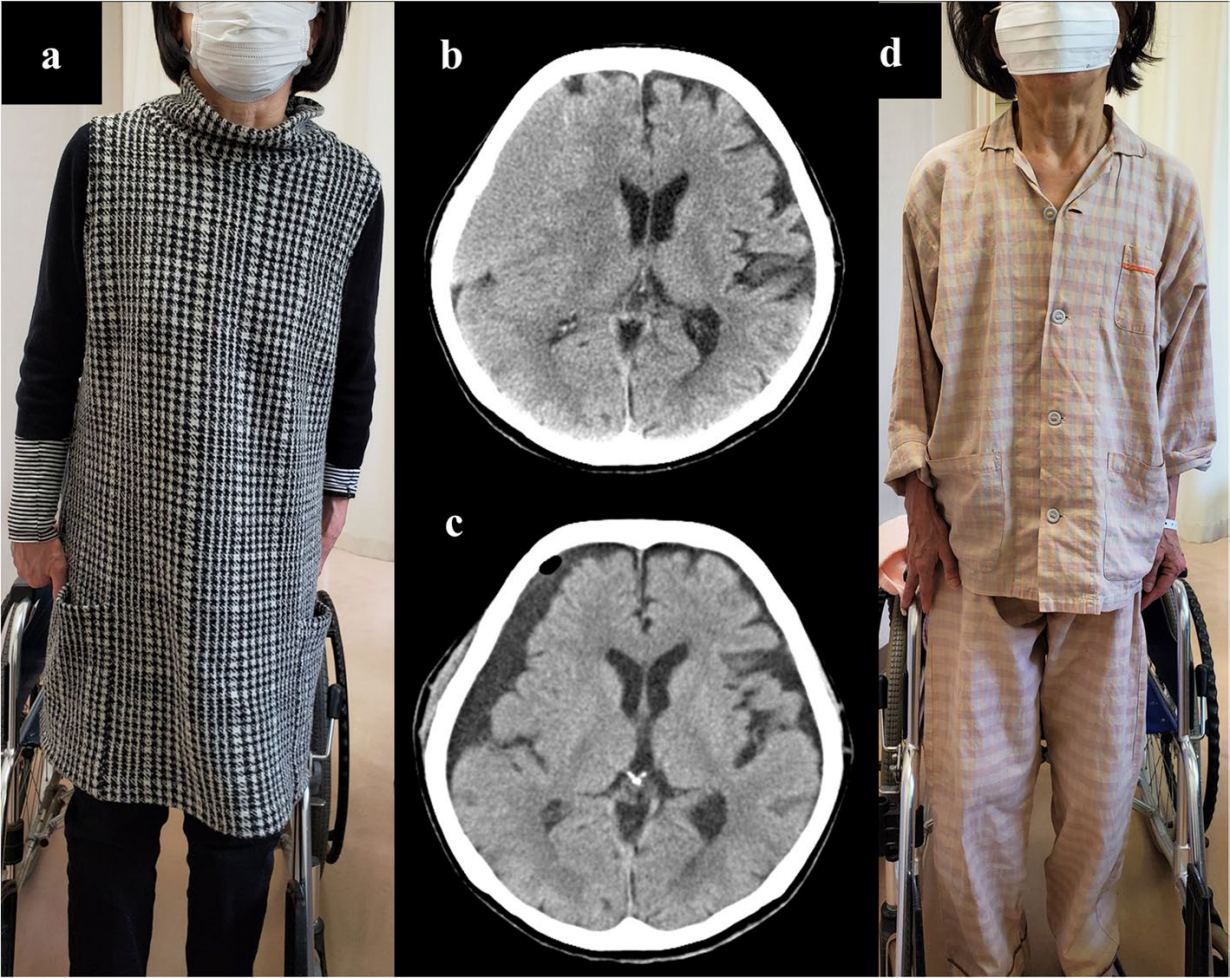
Tonic flexion of the trunk to the left was present in the standing position before surgical intervention (**a**). Presurgical brain CT showing voluminous subdural hematoma in the right convexity with marked compression of the right hemisphere (**b**). Postsurgical brain CT carried out four days after the operation showing improvement of the right subdural hematoma (**c**). Seven days after hematoma evacuation, tonic flexion of the trunk disappeared (**d**)

A 70-year-old woman was diagnosed as having PD at 65 years of age after having difficulty with walking and clumsiness with her hands. She also had olfactory impairment. She had no family history of PD. Examinations revealed asymmetric parkinsonism with limb rigidity and bradykinesia that were more prominent on the left. There were no cerebellar signs or gaze palsy. Her Hoehn and Yahr stage was 2 and her Mini-Mental State Examination score was 30. Magnetic resonance imaging of her brain revealed normal findings. She was initially treated with levodopa (L-dopa), (200 mg/day), which markedly improved her symptoms. A reduced meta-iodobenzylguanidine (MIBG) cardiac uptake on myocardial scintigraphy was detected. On the basis of these findings, she was diagnosed as having PD. At 67 years of age, she developed orthostatic hypotension and was treated sequentially with fluids, compression stockings, and midodrine. Her disease slowly progressed over the next three years, and L-dopa dosage was increased to 600 mg/day. At 70 years of age, she had a mild head trauma with loss of consciousness. One month later, she developed acute-onset lateral flexion of her trunk to the left side while standing, and she was admitted to our hospital. On admission, her neurological examinations revealed slight weakness of her left upper and lower extremities in both proximal and distal muscles, as indicated by her Medical Research Council (MRC) scale score of 5-/5. She showed a sustained 10.1°lateral flexion of her trunk to the left side on standing (Fig. 1a). This posture was alleviated in the supine position. Her Pisa angle was assessed using NeuroPostureApp© (http://www.neuroimaging.uni-kiel.de/NeuroPostureApp) [5]. Her right muscle strength was normal. Her sensory systems were normal and no cerebellar signs were detected. Her deep tendon reflexes were normal and her plantar reflex was flexor bilaterally. Her presurgical brain computed tomography (CT) showed CSDH in the right hemisphere resulting in a marked compression of the hemisphere (Fig. 1b). She was immediately referred to the neurosurgical department of our hospital and the subdural hematoma was successfully evacuated by single-burr-hole drainage. Her brain CT carried out four days after the operation showed improvement of her subdural hematoma (Fig. 1c). Seven days after her operation, her PS disappeared (Fig. 1d) and she fully recovered to her preoperative level of function without any sequelae such as headaches and cognitive impairment caused by her CSDH. She is still being follow-up.

## Discussion and conclusions

PS commonly develops in people with PD, with an estimated prevalence of around 8.8% [6]. The pathophysiology of PD is probably multifactorial, but most data including those from animal and clinical studies support the central hypothesis, rather than the peripheral hypothesis, as shown by the asymmetry of basal ganglia dysfunction and abnormalities in the central integration of sensory information coming from different sources, such as proprioception, the vision and vestibular system, and cognitive dysfunctions affecting the body schema, perception, and postural control [6–8]. PS is more likely to be associated with a significantly longer disease duration, a more severe motor phenotype, ongoing combined treatments with L-dopa and dopamine agonists, and osteoporosis and arthrosis as comorbidities [8, 9]. The majority of patients with PD developed PS chronically, i.e., over a period of more than three months [9], but our patient had an acute onset of PS. Therefore, several other etiologies were considered as the cause of PS, and her brain CT showed CSDH in the right hemisphere. Recently, Marchione et al. have reported the case of a patient with the onset of PS associated with a voluminous unilateral frontal, temporal, and parietal subdural hematoma, which was reversed by evacuation intervention [4]. They suggested the following possible pathophysiology of PS: the basal ganglia compression by a hematoma causes truncal dystonia through thalamocortical trait functional disruption and loss of proprioceptive integration. In addition, it is also considered that a subcortical structural displacement may alter the motor cortex connectivity to the basal ganglia with consequent reduction of inhibition pathways to the sensory–motor cortex.

In our patient, the hematoma in her right hemisphere may have caused the direct mechanical compression of the frontal cortices or the indirect compression of the basal ganglia caused by a midline shift. Takakusaki in his review stated that somatosensory, vestibular, and visual sensations are integrated at the temporoparietal and posterior parietal cortices [10]. The posterior parietal cortex and its connection to the frontal lobe play an essential role in maintaining the body schema [10]. It is possible that the CSDH in our patient impaired the information processing from the temporoparietal cortex to the frontal lobe, including the premotor area and the supplementary motor area for anticipatory postural control. It may also be postulated that the subcortical structural displacement that alters motor cortex connectivity to the basal ganglia contributed to the development of PS [4]. It is considered that the mild weakness in her left upper and lower extremities contributed little to her PS. Further study is required to clarify the pathophysiology of PS.

In conclusion, our case clearly demonstrated that prompt evaluations including neuroimaging are crucial for accurate diagnosis and timely neurosurgical intervention for the complete resolution of acute-onset PS caused by CSDH, even in patients with neurodegenerative disorders such as PD.

## Data Availability

Not applicable.

## Abbreviations

CT: Computed tomography
PD: Parkinson’s disease
PS: Pisa syndrome
CSDH: Chronic subdural hematoma
